# The Effects of a 12-Week Training Multicomponent Exercise Program on Landing Mechanics in Recreational Athletes

**DOI:** 10.3390/healthcare12232327

**Published:** 2024-11-21

**Authors:** Adrián Feria-Madueño, Timothy Hewett, Thomas Dos’Santos, Borja Sañudo

**Affiliations:** 1 Department of Physical Education and Sport, University of Seville, 41013 Sevilla, Spain; bsancor@us.es; 2 Department of Orthopedics, Marshall University Biomechanics Laboratories and Sports Medicine Research, Marshall University, Huntington, WV 25755, USA; hewettt@marshall.edu; 3 Department of Sport and Exercise Sciences, Musculoskeletal Science and Sports Medicine Research Centre, Manchester Metropolitan University, Manchester M15 6BX, UK; t.dossantos@mmu.ac.uk

**Keywords:** landing, kinetic, knee injuries, exercise program

## Abstract

Background/Objectives: This study investigated the impacts of a 12-week training program on kinetic parameters during landings in non-professional recreational athletes. Methods: Fifty-seven non-elite recreational athletes performed three unilateral-landing trials from a 30 cm high structure on a force platform. The following outcome measures were analyzed: the ground reaction forces at initial ground contact (PF1) and the highest value (PF2), impulse (change in the moment of force during landing), stabilization time, and ankle and knee accelerations. The ground reaction forces, momentum, and accelerations were evaluated in the vertical, medio-lateral, and anteroposterior axes. Participants were randomly assigned to two groups. The experimental group (EG) underwent a 12-week intervention, three times per week, consisting of various exercises, such as strength, eccentric, proprioceptive, whole-body vibration (WBV), and neuromuscular exercises. After 12 weeks, the same outcome measures were analyzed. Results: The effects of the training program on vertical ground reaction forces were not clear (0.3% and 0.9%, respectively). Medio-lateral (64.8%, d = 0.51) and anteroposterior (43.9%, d = 1.34) forces were probably reduced due to the training program. The stabilization time was also reduced in the experimental group (44.2%). The training program most likely decreased the vertical impulse (47.3%, d = −1.56), whereas the total ankle acceleration increased (18.4%, d = 0.79). Conclusions: The findings reveal significant improvements in neuromuscular control and stability during landings, specifically demonstrating reduced medio-lateral forces, vertical momentum, and acceleration during monopodal landings. This study focuses on the importance of proper landing techniques in minimizing the risk of knee injuries, emphasizing the falling strategy’s role in injury prevention.

## 1. Introduction

Jump-landing actions are a common injury-inciting event associated with knee injuries. Typically, athletes in sports such as volleyball, basketball, and netball frequently perform jump-landing actions, which can be characterized as complex monopodal actions. Moreover, physically active individuals, irrespective of their specific sport, often engage in a high number of landings, which are primarily characterized as complex monopodal actions. Researchers such as Weinhandl et al. (2010) have linked monopodal landings to a higher injury incidence compared to bipodal landings, most likely attributed to the significantly greater kinetics observed unilaterally compared to bilaterally [1].

The individual’s biomechanical strategy during landings determines the mechanical loading and stress endured by a joint based on the joint structure’s cushioning capacity [2,3]. Thus, the initial contact and early weight acceptance have been identified as moments of greater injury risk [4,5]. However, despite the demonstrated relevance of monopodal landings in increasing the risk of knee and ankle joint injuries, there are different jump-landing assessment methods for these landings, and no consensus exists in this regard. The relationship between kinetics in jumps and their monopodal landings has been explored in the literature [6,7,8]. However, some researchers evaluated landings from 50% of the maximum jump height [9], while other authors have used landing protocols from specific heights, ranging from 20 cm to 60 cm [10] or exactly 28–30 cm [11,12,13,14,15].

In order to achieve a consistent and standardized assessment of landing biomechanics, it is crucial to consider standardizing both the jump height and subsequent monopodal landing, as these factors play a pivotal role in understanding the injury mechanism during landings [14,16,17,18]. Moreover, it is pertinent to identify which variables contribute to a higher risk of knee injuries, including factors such as muscle activation, knee abduction, internal rotation, increased peak force, and degree of knee extension during landing moments [8,19,20,21,22]. All these factors are amplified when analyzing monopodal landings [23].

During jump-landing actions, understanding specific biomechanical strategies is crucial. Previous studies have described the impacts of implementing training programs on landing mechanics, with the potential to significantly enhance athletic performance and reduce the risk of lower-limb injuries.

Sasaki et al. reported a positive effect on landing kinetics following an 8-week training program, where the peak knee-valgus moment decreased from the initial jump [24]. One of the primary limitations was their exclusive focus on female subjects, with the sole kinetic variable evaluated being the moment of force. Probably, the polarization towards the female sex responds to a reality where women manifest a higher risk of knee injuries than men. In another recent study, Otsuki et al. investigated the effects of a preventive training program on jump landings in relation to the athletes’ maturation level [25]. It appears that interventions through preventive training programs in post-pubertal ages may serve as highly reliable strategies for establishing proper movement patterns that reduce the risk of knee injuries during landings. A recent comprehensive systematic review demonstrated the effects of best-practice preventive training guidance on ACL in female athletes [26]. As the main conclusion, the authors recommended that training programs should focus on preventive neuromuscular training, including strength exercises and jump stabilization actions. Specifically, a recent meta-analysis explained the effects of preventive training programs on biomechanical aspects during landing actions [27]. They supported the role of preventive programs in reducing injury risks but suggested that these programs could be enhanced by targeting participants’ baseline profile deficits through individualized assessments before participation in the training program.

Despite finding contributions in the literature regarding the effects of training on reducing the risk of knee injuries during jump landings, there is a notable bias towards female athletes in these programs, and there is a shortage of evaluations of training program effects on multiple kinetic variables during landings. Consequently, the aim of the present study was to analyze the effects of a 12-week training program on kinetic parameters during landings in non-professional recreational athletes. The hypothesis of the present study is that after the training program, strength in the medio-lateral (ML) and anteroposterior (AP) axes will be reduced, in addition to stabilization time (TdEst), impulse, and acceleration.

## 2. Materials and Methods

### 2.1. Participants

Fifty-seven non-elite recreational athletes (age = 23.01 ± 2.99 years; mass 73.01 ± 12.76 Kg; height 1.74 ± 0.08 m; 23% female) were recruited for this study. All participants engaged in physical exercise based on outdoor running, team sports such as soccer and basketball, and fitness-room exercises (physical activity per week = 8.78 (±4.30) hours). Individuals with injuries that prevented them from completing the test protocol were excluded from participating. Before the motion analysis laboratory test, all participants underwent a structured interview to assess the number and severity of lower-limb injuries sustained in the past 12 months, their playing experience, and their involvement in systematic resistance training (more than two sessions per week). Additionally, they refrained from exercising 48 h prior to the test. Informed verbal consent was obtained from all participants, and written consent was provided before their inclusion in the study. This study received approval from the Ethics Committee of the respective institution and is in accordance with the Declaration of Helsinki.

### 2.2. Experimental Design

A randomized controlled trial intervention study with a repeated-measures design was conducted to evaluate the impacts of a 12-week tailored exercise program on kinetic activity during landing tasks. The participants were randomly assigned to an experimental group (EG, *n* = 29) and a control group (CG, *n* = 28). Randomization was performed by a team member external to the evaluation and recruitment, using the OxMaR 1.0 software [28], ensuring allocation concealment. The randomization was stratified by gender to ensure a balanced representation of males and females in both the experimental and control groups. The sample size was calculated using a *t* test for differences between independent groups. Through a pilot study using the G*power software (v3.1, Heinrich-Heine-University, Dusselford, Germany), the required sample for both groups was determined to be 26 participants, aiming for an effect size of 0.8, a α of 0.05, and a power of 0.80. This calculation allowed for potential dropouts to maintain statistical power. Baseline assessments were performed on both groups, including three maximal isometric actions of the quadriceps and hamstrings in the laboratory, with the maximal voluntary contraction (MVC) being evaluated by a Biodex System isokinetic dynamometer. The next day, each subject underwent a landing test with 3 landing trials.

### 2.3. Procedures

**Figure 1:**
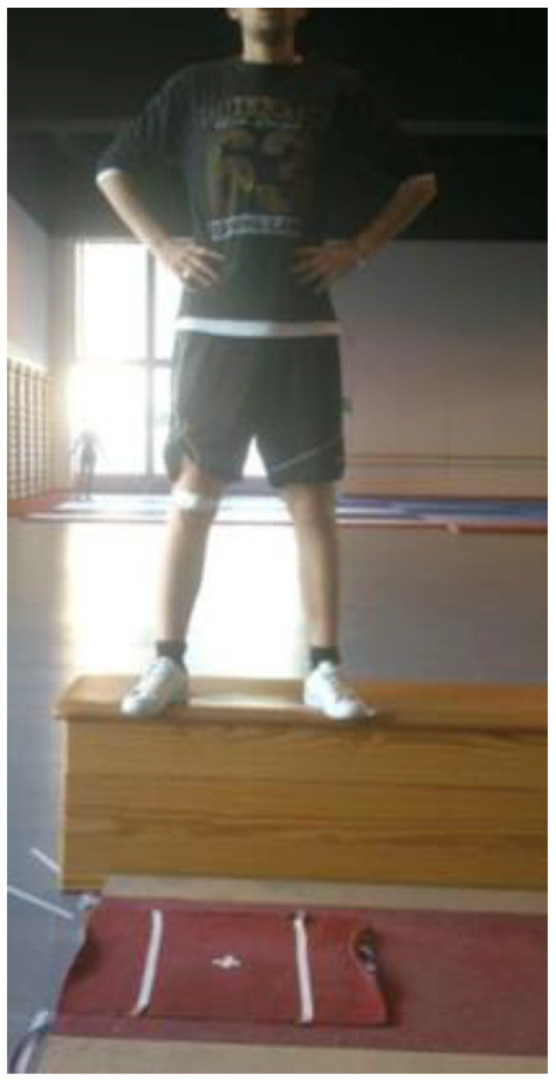
Participant with hands on hips preparing to perform a single leg landing as part of the experimental protocol.

After a 5 min warm-up period, participants familiarized themselves with the sports-specific landing tasks. Participants performed 3 unilateral-landing trials (with a 1 min rest period between each attempt) from a 30 cm high structure on a force platform (Figure 1). To standardize the attempts, subjects were required to keep their arms resting on their hips and their gaze straight ahead, keeping their feet shoulder-width apart. No specific instructions were provided regarding the landing strategy, and the only information provided was that they were to land with their dominant foot dropping forward.

Following the baseline assessment, participants were randomly assigned to either the EG or CG. Throughout the 12-week intervention period, all participants continued with recreational sports, and their physical activity was assessed before and after the intervention. The participants continued to practice their daily physical activity 3 times per week during the 12 weeks of the program. The EG underwent a 12-week intervention, three times per week, consisting of various exercises, such as strength, eccentric, proprioceptive, whole-body vibration (WBV), and neuromuscular exercises (Supplement S1). These exercises were designed by experienced sports scientists, and their complexity increased over the weeks. The compliance rate for each participant was more than 80% of the sessions, i.e., a minimum of 29 completed sessions was considered for the post-test. During the initial three weeks, the training sessions lasted approximately 40 to 45 min and encompassed exercises involving WBV, proprioception, eccentric strength, suspension training, and balance training. In the subsequent three weeks (weeks four, five, and six), the same set of exercises was performed but with a variation in their combination (e.g., WBV and balance exercises) within each 50 min session. Over the following three weeks, the program was further enhanced by introducing exercises involving cross-jumps and coordination tasks. This phase saw an increase in the total number of exercises, sets, and repetitions, resulting in a total session duration of 60 min. All sessions were supervised by an independent sports scientist not involved in the research. The CG continued their regular exercise routine. After 12 weeks, both the EG and the CG underwent evaluation in the same laboratory, following the same procedures as the baseline assessment.

### 2.4. Data Processing—Isometric Testing

Participants performed three MVCs for the hamstring and quadricep muscles, and the forces were normalized to their body weight. A Biodex System isokinetic dynamometer (Biodex Multi-joint System, Shirley, New York, NY, USA), with a sampling rate of 1000 Hz, was used. The three MVC tests were performed in a seated position, with a 3 min rest between attempts. To assess the hamstring muscles, the knee was flexed to 45 degrees [29] by the Biodex System (ICC = 0.79). For the evaluation of the quadriceps (ICC = 0.89), a seated position with the knee extended at 45 degrees was used [30]. All participants wore a trunk harness to restrict movement. The MVC test was executed by aligning the subject’s system with their dominant leg, facilitating mechanical action along the AP axis. The investigators provided verbal feedback to encourage maximal effort during isometric contractions, which were held for 5 s.

### 2.5. Kinetics

A triaxial force platform was employed to assess landing tasks (Kistler 9260 AA6, Winterthur, Switzerland) set at a sampling rate of 1000 Hz. The primary variables analyzed through the force platform encompassed ground reaction forces (GRFs), measured at two different points in time: initial ground contact (PF1) and when the highest force value was reached (PF2). TdEst, and impulse, were evaluated using the force plate. Moreover, knee and ankle accelerations during landings were assessed using two triaxial accelerometers (xyzPLUX; PLUX—Wireless Biosignals, Lisbon, Portugal). One of these accelerometers was affixed to the lateral condyle and the other to the lateral malleolus using adhesive tape for precise measurement. The signals underwent preprocessing to eliminate the impact of gravitational forces.

### 2.6. Outcome Measures

Kinetic variables through the force platform and by accelerometry were evaluated. PF1 was evaluated as the vertical force value at the first contact with the ground after landing. PF2 was analyzed as the maximum peak vertical force. The highest force value reached in the medio-lateral (ML) and anteroposterior (AP) axes was also evaluated. Thus, the force in the ML axis had two values, one positive (Force_ML+_) and the other negative (Force_ML−_), as well as the force in the AP axis (Force_AP+_ and Force_AP−_, respectively). In the ML axis, the positive sign indicates the force toward the dominant side (right in right-handers and left in left-handers), and in the AP axis, the positive sign signifies the force exerted toward the front, and the negative sign toward the back. TdEst is described as the eccentric contact time (seconds). Momentum is defined as the change in the moment of force during landing. This variable was also evaluated on the vertical axis (IZ), on the AP axis (IAP), and on the ML axis (IML).

Total accelerations were evaluated both at the ankle joint (ACC_ANKLE_TOTAL) and at the knee joint (ACC_KNEE_TOTAL). Similarly, accelerations in partials were analyzed for both joints, both for the ML axis (ACC_ANKLE_ML and ACC_KNEE_ML, respectively); in the AP axis (ACC_ANKLE_AP and ACC_KNEE_AP, respectively); and in the vertical axis (ACC_ANKLE_Z and ACC_KNEE_Z, respectively).

### 2.7. Statistical Analysis

A statistical analysis was performed using the SPSS statistics software version 25.0. The Kolmogorov–Smirnov test, independent *t*-tests, and repeated-measure two-way ANOVAs (time × group) were utilized. The level of significance was set at *p* ≤ 0.05. The data are presented as mean values along with their standard deviations (mean ± SD), and Intraclass Correlation Coefficients (ICCs) were computed. Additionally, the effect size (ES) was determined with corresponding 90% confidence limits (90% CI) for the variables under investigation, and Cohen’s d was determined. A qualitative assessment of potential quantitative changes resulting from the program was conducted as follows: changes of less than 1% were considered almost certainly not significant, changes in the range of 1–5% were deemed very unlikely to be significant, changes between 5–25% were categorized as unlikely to be significant, changes from 25–75% were regarded as possible, changes within the range of 75–95% were considered likely, changes of 95–99% were seen as very likely, and changes exceeding 99% were categorized as almost certain [31]. Finally, to correct for multiple comparisons of all variables analyzed, a Tukey test was performed (Supplement S2).

## 3. Results

Intragroup changes were analyzed after the 12-week intervention (Table 1). Although the effects on both PF1 (0.3% [90% CI: −6.7]; 7.9) and PF2 (0.9% [90% CI: −4.7]; 6.8) were not clear, the reduction in values of Lateral Force+ (−49.4% [90% CI: −58.3]; −38.6); Lateral Force− (−64.8% [90% CI: −77.5]; −44.9); Anterior–Posterior Force+ (−43.9% [90% CI: −54.1]; −31.5); Anterior–Posterior Force− (−39.6% [90% CI: −50.1]; −27.1); TdEst (−44.2% [90% CI: −49.9]; −37.9); Impact Zone (−47.3% [90% CI: −54.3]; −39.3); and Impulse in the Anterior–Posterior direction (−61.9% [90% CI: −73.1]; −46.0) were certainly due to the training program in the EG. Regarding acceleration, both ankle and knee acceleration increased in all axes after training in the EG. However, the change produced in ACCKNEEML was not clear (−5.7% [90% CI: −45.5]; 63.0). As for the CG, it can be observed that PF1 probably increased after the 12-week period (14.8% [90% CI: 1.6]; 29.8). However, the increase in PF2 was not clear (4.1% [90% CI: −0.8]; 9.3). Nevertheless, there was an average increase of 0.34 s in TdEst, although the effect of time on this increase was not clear (12.0% [90% CI: −8.1]; 36.5).

Changes between groups were studied (Table 2). The training program likely influenced PF1 to remain constant in the EG compared to the CG, where this variable increased by 2.3 N (0.3% [90% CI: −6.7]; 7.9). Only Lateral Force− (varus) was reduced, probably due to training (−49.4% [90% CI: −58.3]; −38.6). However, TdEst was certainly reduced by the training program in the EG compared to the CG by 0.93 s, showing significant differences between the two groups after 12 weeks (*p* = 0.008). A reduction in the Impact Zone (IZ) likely due to training was observed in the EG compared to the CG (−47.3% [90% CI: −54.3]; −39.3), with significant differences between the groups (*p* = 0.038). Additionally, Impulse Moment Lateral (IML) also decreased, although to a lesser extent or possibility. On the other hand, the Total Ankle Acceleration (ACC_ANKLE_TOTAL) likely increased due to the training program in the EG compared to the CG (−18.4% [90% CI: −29.5]; −5.6). Furthermore, Inward Moment Lateral (ACC_ANKLE_ML) and Inward Moment Anterior–Posterior (ACC_ANKLE_AP) also increased in the EG due to the intervention program, although the effect was not clear. The effect on Inward Moment Vertical (ACC_ANKLE_Z) was stronger, and it is very likely that the training program was responsible for a 1.33-point increase in this parameter in the EG (−50.8% [90% CI: 29.4]; 75.6). However, no significant differences were found between the groups (*p* > 0.05).

Again, with respect to Ankle Acceleration Vertical (ACC_KNEE_Z), this increased by 2.59 points in the EG, most likely due to the training program, but no intergroup differences were found (*p* > 0.05).

**Figure 2:**
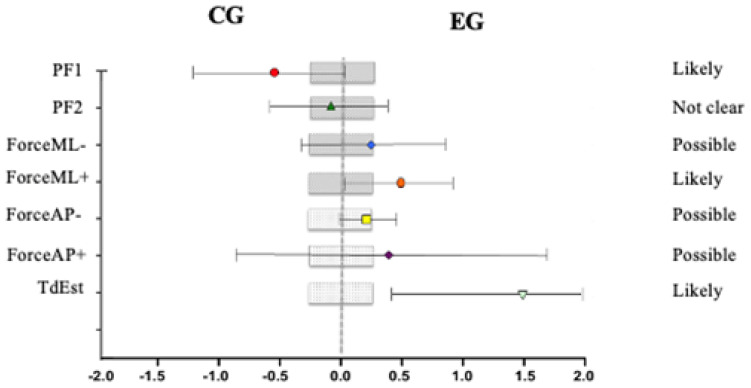
Comparative effects of the training program in both groups in relation to kinetic variables during landings. PF1 (N/Kg) = ground reaction force at first contact with the ground. PF2 (N/Kg) = peak maximum vertical force during landing. ML− force (N/Kg) = force in the medio-lateral axis exerted in varus. ML+ force (N/Kg) = force in the medio-lateral axis exerted in valgus. Force_AP−_ (N/Kg) = force in the anterior–posterior axis exerted backward. Force_AP+_ (N/Kg) = force in the anterior–posterior axis exerted forward. TdEst (s) = stabilization time after landing. Bars indicate changes in means with a 90% confidence interval.

The effect size of the training program on kinetic parameters during landings is shown in Figure 2. Graphically, it can be observed that the intervention’s effect on PF1 is likely to result in maintaining the value in the EG compared to an increase in the CG. Similarly, both Lateral Force+ in valgus and TdEst are likely to improve in the EG compared to the CG due to the intervention.

**Figure 3:**
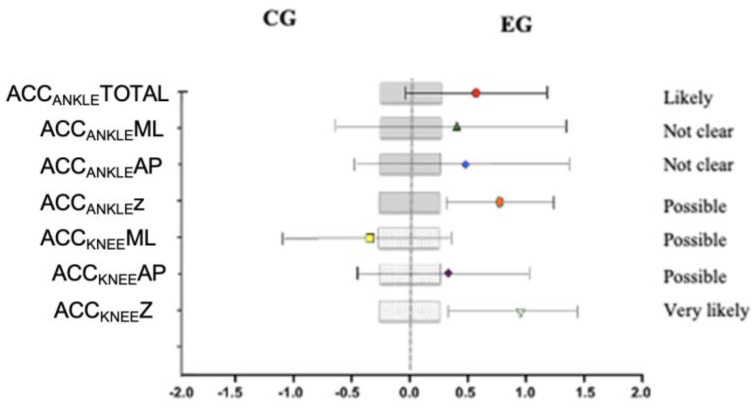
Comparative effects of the training program in both groups in relation to the kinetic variables of acceleration during landings. ACC_ANKLE_TOTAL (g) = total localized acceleration at the ankle during ground contact. ACC_ANKLE_ML (g) = partial acceleration of the ankle in medio-lateral axis when GRF is maximum. ACC_ANKLE_AP (g) = partial acceleration of the ankle in anterior–posterior axis when GRF is maximum. ACC_ANKLE_Z (g) = partial acceleration of the ankle in vertical axis when GRF is maximum. ACC_KNEE_ML (g) = partial acceleration of the knee in medio-lateral axis when GRF is maximum. ACC_ANKLE_AP (g) = partial acceleration of the knee in anterior–posterior axis when GRF is maximum. ACC_ANKLE_Z (g) = partial acceleration of the knee in vertical axis when GRF is maximum.

Furthermore, changes in acceleration during landings were also analyzed graphically, and these changes can be observed in Figure 3. Overall, both the ankle and knee acceleration improved in favor of the EG compared to the CG, possibly or probably due to the training.

## 4. Discussion

The present study aimed to analyze the effects of a 12-week training program on kinetic parameters during landings in non-professional recreational athletes. The main finding of this study is that after the training program in this population, there was a reduction in medio-lateral forces, vertical momentum, and acceleration during monopodal landings, which was related to a reduction in the risk of knee injuries. In relation to the landing technique, the falling strategy appears to be important in reducing the risk of injury [32,33]. To control differences between landing techniques, all subjects were asked to perform single-foot landings with their hands on their hips, without providing further information on the ideal landing form. This restriction on information was implemented to avoid contaminating the landing strategies. According to Heinert et al. [34], the instructions given to a subject for performing a landing are crucial in determining the kinetic outcomes of the sports gesture, and they could even contribute to significantly reducing GRFs. However, in the present study, neither PF1 nor PF2 differed significantly before and after the training program. Although similar values of PF1 and PF2 have been reported in the literature as traits related to knee injury incidences [35], subjects who did not undergo training (CG) experienced an increase in PF1, probably due to the passage of the 12 weeks (d = 0.49). This fact seems to be directly related to the likelihood of knee injuries during landings for the CG, suggesting that the training program may have served to safeguard PF1 in the EG, as it remained practically stable before and after 12 weeks of training (d = 0.01). According to Pflum et al. [36], high peak forces during ground contacts after landing imply an increase in the load supported by the anterior cruciate ligament (ACL) of the knee; however, it is not clear whether exercise programs such as the one developed in this study can contribute to maintain these loads and ensure the described values through peak forces related to the incidence of injury during landings. A possible explanation may be that despite high force values, the activation of the anterior and posterior thigh musculature is enhanced, achieving neuromuscular adaptations that help to improve knee stabilization during landings [19]. However, muscle activation was not assessed in this study.

Regarding lateral (varus) landing forces, these were also reduced, probably due to the training (d = −0.51). As indicated by Zebis et al. [37], preventive training could reduce the risk of knee injuries by improving neuromuscular control during ground contact, including the frontal plane. A contributing factor that supports this fact may be the reduction in TdEst after landing (decrease of 0.93 s), certainly due to the training program (d = −2.15). A lower TdEst may indicate better muscular control during landings, which is related to greater joint stability [38].

On the other hand, although precise data on vertical ground reaction forces (VGRFs), lateral forces, and anterior–posterior forces were recorded, the recent literature associates knee injuries with variations in force moments during landings [39,40]. This force moment provides information about the shear force that occurs in the knee during these types of movements, which is of great importance, since a clear relationship has been demonstrated between the varus or valgus rotation of the limb supporting the ground and an increased risk of knee injuries [41]. Additionally, the mechanical impulse, which represents the change experienced by this force moment, was also analyzed as one of the main novelties of the present study. In the literature, there are some examples, such as Krupenevich et al. [42], where the moment and impulse are related, with an emphasis on the net joint moment and the change during ground contact through impulse.

When analyzing these variables during landings, it was observed that the training program most likely decreased the impulse in the vertical direction (d = −1.56 [−2.42; −0.71]). According to Mojaddarasil et al. [43], a reduction in impulse during landings could translate into a significant decrease in the risk of knee injuries. This reduction could be related to a decrease in shear or compressive forces during monopodal landings, since, according to numerous authors, compressive vertical forces accompanied by rotations during landings could be responsible for the increased risk of injury [23,44,45,46]. Despite all this, and according to the consulted literature, it seems that this is the first study to analyze the impulse before and after carrying out a training program and evaluate it through monopodal landings in physically active subjects.

## 5. Limitations

The present study is not without limitations that undoubtedly need to be considered. The first of these limitations is the absence of a critical analysis of the risk of knee injuries using kinematic variables. Despite having detailed multiple kinetic variables that describe not only the ground reaction forces but also knee and ankle acceleration, as well as stabilization time, a kinematic evaluation was not conducted. This omission may have limited the specific information on variables related to knee injuries during landings, as the literature indicates that kinematic responses can be highly correlated with the biomechanics of shock absorption during landings.

Nevertheless, the present study focused on the described kinetic variables that explain the landing maneuver from a mechanical causation perspective, rather than describing the maneuver itself. Future studies should integrate a kinematic perspective to complement the main kinetic findings, thereby ensuring a more comprehensive view of the biomechanical analysis of the action.

## 6. Conclusions

The 12-week training program conducted with non-professional recreational athletes showed significant improvements in kinetic parameters associated with landings, with notable changes reflecting better force control and optimized stabilization post-impact. Although changes in the vertical ground reaction forces were minimal, the program significantly reduced forces in the medio-lateral (ML) and anteroposterior (AP) directions, suggesting superior control of forces in those directions. Additionally, the reduction in stabilization time highlights an improvement in postural control after landing, a critical factor for injury prevention in impact sports or activities involving jump landings. The decrease in vertical impulse and the increase in ankle acceleration also reflect neuromuscular adaptation, indicating a greater capacity of the lower limbs to absorb impact efficiently, which could be relevant in reducing knee injury risks.

Since these results show a positive trend in optimizing landing mechanics, future research should consider extending the duration and frequency of training, exploring the effects of longer programs on injury risk reduction. Furthermore, it would be beneficial to assess the effectiveness of this training in other groups, such as higher-level athletes or athletes from different sports disciplines to determine if these benefits can be generalized and to explore comparisons across sports. Another important aspect to explore is the adaptation of these programs to sport-specific contexts by integrating functional exercises that mimic specific movement patterns.

Finally, incorporating analyses of psychological factors, such as landing confidence and perceived injury risks, could provide a comprehensive view of how this type of training not only impacts physical performance but also enhances athletes’ perceived safety. This could lead to stronger adherence to preventive programs among recreational and professional populations.

## Figures and Tables

**Table 1:** Intragroup changes in kinetic parameters in landings after the training program in the EG and CG.

	Pre		Post		GE		CG	
**Variables**	**EG**	**CG**	**EG**	**CG**	**Cohen’s d**	**Change**	**Cohen’s d**	**Change**
PF1 (N/Kg)	18.29 (5.68)	15.97 (4.02)	18.30 (5.27)	18.27 (5.37)	0.01 (−0.24; 0.26)	11/81/8%	0.49 (0.06; 0.92)	87/13/1%
PF2 (N/Kg)	47.97 (8.30)	45.07 (7.36)	48.44 (9.30)	46.80 (6.80)	0.05 (−0.25; 0.34)	19/73/8%	0.24 (−0.05; 0.53)	59/35/6%
Force_ML+_ (N/Kg)	9.31 (3.43)	9.03 (2.59)	4.41 (1.22)	4.52 (1.10)	−1.14 (−1.47; −0.82)	0/0/100%	−2.31 (−2.60; −2.02)	0/0/100%
Force_ML−_ (N/Kg)	1.20 (0.97)	0.94 (0.85)	0.40 (0.33)	0.63 (0.40)	−0.90 (−1.29; −0.52)	0/0/100%	−0.51 (−0.89; −0.12)	0/9/91%
Force_AP+_ (N/Kg)	2.59 (1.29)	2.51 (1.24)	1.21 (0.54)	1.35 (0.44)	−0.82 (−1.11; −0.54)	0/0/100%	−1.34 (−1.76; −0.92)	0/0/100%
Force_AP−_ (N/Kg)	2.62 (1.63)	2.85 (1.43)	1.45 (0.78)	1.59 (0.69)	−0.68 (−0.94; −0.43)	0/0/100%	−0.97 (−1.34; −0.60)	0/0/100%
TdEst (s)	2.06 (0.46)	1.98 (0.48)	1.13 (0.14)	2.32 (1.10)	−2.48 (−2.94; −2.03)	0/0/100%	0.44 (−0.33; 1.20)	70/22/8%
I_z_ (N × s)	21.97 (6.73)	20.92 (5.22)	11.52 (3.12)	20.45 (9.21)	−2.13 (−2.61; −1.66)	0/0/100%	−0.34 (−0.94; 0.27)	7/28/65%
I_AP_ (N × s)	0.20 (0.25)	0.21 (0.25)	0.05 (0.03)	0.09 (0.18)	−0.94 (−1.28; −0.60)	0/0/100%	−1.31 (−1.86; −0.77)	0/0/100%
I_ML_ (N × s)	0.79 (0.33)	0.78 (0.23)	0.35 (0.05)	0.36 (0.09)	−0.45 (−0.77; −0.13)	0/10/90%	−1.96 (−2.52; −1.40)	0/0/100%
ACC_ANKLE_TOTAL (g)	6.56 (1.45)	8.36 (0.79)	8.09 (2.94)	8.91 (3.72)	−0.79 (−1.35; −0.22)	0/4/96%	0.26 (−0.66; 1.19)	55/25/20%
ACC_ANKLE_ML (g)	4.15 (1.04)	4.93 (0.59)	4.40 (3.59)	5.56 (4.64)	−0.40 (−1.23; 0.43)	11/23/66%	−0.07 (−1.18; 1.05)	34/24/42%
ACC_ANKLE_AP (g)	3.35 (1.28)	4.72 (0.97)	4.16 (1.01)	4.23 (0.77)	0.50 (0.03; 0.97)	86/14/1%	0.08 (−0.26; 0.41)	27/65/9%
ACC_ANKLE_Z (g)	3.53 (1.28)	4.73 (0.61)	4.86 (0.24)	4.64 (0.99)	0.82 (0.52; 1.13)	100/0/0%	−0.53 (−1.72; 0.67)	15/17/68%
ACC_KNEE_ML (g)	2.78 (1.69)	4.65 (1.36)	3.10 (2.69)	4.38 (1.57)	−0.06 (−0.65; 0.52)	23/43/35%	−0.42 (−0.98; 0.13)	3/13/75%
ACC_KNEE_AP (g)	1.65 (1.38)	4.36 (1.47)	2.58 (1.59)	3.81 (1.44)	0.44 (/0.04; 0.92)	80/18/2%	−0.39 (−0.66; −0.11)	0/13/87%
ACC_KNEE_Z (g)	2.15 (1.67)	4.62 (0.95)	4.74 (0.48)	4.51 (1.11)	0.94 (0.62; 1.26)	100/0/0%	−0.12 (−0.65; 0.41)	16/45/40%

Abbreviations: CL: confidence level. Note: All differences are presented as improvements (positive), so that positive and negative differences are in the same direction. PF1 (N/Kg) = ground reaction force at first ground contact. PF2 (N/Kg) = peak maximum vertical force during landing. ML− force (N/Kg) = force in the medio-lateral axis exerted in varus. ML+ force (N/Kg) = force in the medio-lateral axis exerted in valgus. Force_AP−_ (N/Kg) = force in the anterior–posterior axis exerted backwards. Force_AP+_ (N/Kg) = force in the anterior–posterior axis exerted forward. TdEst (s) = stabilization time after landing. I_z_ (N × s) = impulse exerted on the vertical axis. Impulse_AP_ (N × s) = impulse exerted on the anterior–posterior axis. Impulse_ML_ (N × s) = impulse exerted on the medio-lateral axis. ACC_ANKLE_TOTAL (g) = total localized acceleration at the ankle during ground contact. ACC_ANKLE_ML (g) = partial acceleration of the ankle in medio-lateral axis when GRF is maximum. ACC_ANKLE_AP (g) = partial acceleration of the ankle in anterior–posterior axis when GRF is maximum. ACC_ANKLE_Z (g) = partial acceleration of the ankle in vertical axis when GRF is maximum. ACC_KNEE_ML (g) = partial acceleration of the knee in medio-lateral axis when GRF is maximum. ACC_KNEE_AP (g) = partial acceleration of the knee in anterior–posterior axis when GRF is maximum. ACC_KNEE_Z (g) = partial acceleration of the knee in vertical axis when GRF is maximum.

**Table 2:** Intergroup changes in kinetic parameters after 12 weeks: EG vs. CG, regarding landing.

Variables	Intergroup % (95% CL) *p* ^a^	Changes	
		% (90% CL)	Change
PF1 (N/Kg)	0.109	0.3 (−6.7; 7.9)	3/14/83%
PF2 (N/Kg)	0.536	0.9 (−4.7; 6.8)	13/47/39%
Force_ML+_ (N/Kg)	0.102	−49.4 (−58.3; −38.6)	3/25/72%
Force_ML−_ (N/Kg)	0.820	−64.8 (−77.5; −44.9)	5/18/77%
Force_AP+_ (N/Kg)	0.795	−43.9 (−54.1; −31.5)	44/38/17%
Force_AP−_ (N/Kg)	0.967	−39.6 (−50.1; −27.1)	41/41/18%
TdEst (s)	0.008 *	−44.2 (−49.9; −37.9)	0/0/100%
I_z_ (N × s)	0.038 *	−47.3 (−54.3; −39.3)	0/0/99%
I_AP_ (N × s)	0.112	−61.9 (−73.1; −46.0)	44/29/28%
I_ML_ (N × s)	0.324	−41.0 (−59.4; −14.1)	74/20/6%
ACC_ANKLE_TOTAL (g)	0.202	−18.4 (−29.5; −5.6)	86/12/3%
ACC_ANKLE_ML (g)	0.098	−14.1 (−37.2; 17.4)	65/20/15%
ACC_ANKLE_AP (g)	0.067	26.9 (1.4; 58.9)	70/18/11%
ACC_ANKLE_Z (g)	0.211	50.8 (29.4; 75.6)	97/3/0%
ACC_KNEE_ML (g)	0.322	−5.7 (−45.5; 63.0)	11/25/65%
ACC_KNEE_AP (g)	0.425	62.2 (−4.0; 173.9)	64/25/11%
ACC_KNEE_Z (g)	0.222	258.6 (133.1; 451.6)	98/2/0%

Abbreviations: CL: confidence level. ^a^ Differences in changes between groups. Note: All differences are presented as (positive) improvements, so that positive and negative differences are in the same direction. PF1 (N/Kg) = ground reaction force at first ground contact. PF2 (N/Kg) = peak maximum vertical force during landing. ML− force (N/Kg) = force in the medio-lateral axis exerted in varus. ML+ force (N/Kg) = force in the medio-lateral axis exerted in valgus. Force_AP−_ (N/Kg) = force in the anterior–posterior axis exerted backwards. Force_AP+_ (N/Kg) = force in the anterior–posterior axis exerted forward. TdEst (s) = stabilization time after landing. I_z_ (N × s) = impulse exerted on the vertical axis. Impulse_AP_ (N × s) = impulse exerted on the anterior–posterior axis. Impulse_ML_ (N × s) = impulse exerted on the medio-lateral axis. ACC_TOBILLO_TOTAL (g) = total localized acceleration at the ankle during ground contact. ACC_ANKLE_ML (g) = partial acceleration of the ankle in medio-lateral axis when GRF is maximum. ACC_ANKLE_AP (g) = partial acceleration of the ankle in anterior–posterior axis when GRF is maximum. ACC_ANKLE_Z (g) = partial acceleration of the ankle in vertical axis when GRF is maximum. ACC_KNEE_ML (g) = partial acceleration of the knee in medio-lateral axis when GRF is maximum. ACC_KNEE_AP (g) = partial acceleration of the knee in anterior–posterior axis when GRF is maximum. ACC_KNEE_Z (g) = partial acceleration of the knee in vertical axis when GRF is maximum. * *p* ˂ 0.05.

## Data Availability

The original contributions presented in this study are included in the article/Supplementary Material, and further inquiries can be directed to the corresponding author.

## Supplementary Materials

The following supporting information can be downloaded at: https://www.mdpi.com/article/10.3390/healthcare12232327/s1. Supplement S1: description of the training program. Supplement S2: Tukey’s test.
