# Supplementary material for: The Effects of a 12-Week Training Multicomponent Exercise Program on Landing Mechanics in Recreational Athletes
Source: Healthcare (Basel). 2024 Nov 21;12(23):2327. doi: 10.3390/healthcare12232327 (PMC11641551; doi:10.3390/healthcare12232327)
Supplement: Supplementary file 1 [file healthcare-12-02327-s001.zip › healthcare-3287329-supplement S1 - Description of the training program.pdf]

Supplement S1

Description of the training program

Week 1

This program begins with a 40-minute routine featuring whole-body vibration exercises on a platform (30Hz-4mm) and proprioceptive activities on a balance platform. It also includes eccentric training with a Russian belt without added weight and suspension exercises using a TRX® system. Finally, there's proprioceptive work using a fitball. Each exercise is performed in 1 series of 10 repetitions with a 1-minute rest between sets.

Week 2

In the second week, the duration increases to 45-50 minutes. Whole-body vibration exercises are maintained with 1 series of 20 repetitions and a 1-minute rest between sets. Proprioceptive exercises on the balance platform are performed with closed eyes, and eccentric training now includes an external load (~10% BMI) with 1 series of 10 repetitions. Suspension training and proprioceptive fitball exercises remain at 1 series of 10 repetitions, with some exercises increasing to 12 repetitions.

Week 3

This week focuses on incorporating jumps into the vibration and proprioceptive exercises, along with coordination training using an agility ladder. Eccentric exercises are done with Therabands, and fitball balance exercises are also included. Sessions last 45-50 minutes with each exercise performed for 1 series of 10 repetitions and a 1-minute rest.

Week 4

Whole-body vibration and proprioceptive exercises continue with jumps from the previous week, and the vibration exercise volume increases to 20 repetitions. Coordination exercises with the agility ladder continue, with eccentric training performed with Therabands at 1 series of 16 repetitions. Fitball balance exercises are included, with a session duration of 45-50 minutes.

Week 5

Sessions extend to 50-55 minutes, introducing proprioceptive exercises with turns on the balance platform and coordination work with the agility ladder. Eccentric exercises are done with Therabands and a foam ball, performed with closed eyes for some tasks. Fitballs and other proprioceptive tools are used, with each exercise at 1 series of 10 repetitions and a 1-minute rest.

Week 6

This week combines vibration and balance platforms for whole-body vibration exercises. Cross-jumps on the balance platform and isometric training with a Russian belt on the balance platform are included. Suspension and proprioceptive exercises use elements like fitballs, balance platforms, and foam balls for a session lasting 50-55 minutes, each exercise at 1 series of 10 repetitions.

Week 7

Routine variations include closed-eye vibration exercises and cross-jumps. Eccentric training with a Russian belt and external load is performed on the balance platform. Suspension exercises with the TRX® system and coordination with the agility ladder are included, all done at 1 series of 16 repetitions with a 1-minute rest for each exercise.

Week 8

Proprioceptive exercises include closed-eye cross-jumps and balance work. Equipment such as the trampoline and agility ladder are used, along with balance exercises on fitballs and platforms. The session duration is 50-55 minutes, with each exercise done in 1 series of 10 repetitions.

Week 9

Proprioceptive and balance exercises are emphasized, with some done with closed eyes. Trampoline and balance platforms are used together, with coordination training on the agility ladder. The session duration is 55-60 minutes, with 1 series of 10 repetitions per exercise.

Week 10

Proprioceptive exercises include turns on the balance platform and complex movements like inverted balance. Eccentric training with Therabands and

trampoline is reinforced, along with coordination and balance using the agility ladder. Sessions last 55-60 minutes, with 1 series of 10 repetitions.

Week 11

This phase incorporates proprioceptive and balance exercises with jumps and turns, using the trampoline and balance platforms. Eccentric exercises with Therabands and foam elements, plus agility ladder coordination, are maintained for 1 series of 10-20 repetitions. Sessions are 55-60 minutes.

Week 12

In the final week, stop tasks are added to proprioceptive exercises, with continued use of trampoline, balance platforms, and Therabands. Coordination exercises involve jumps and complex moves with the agility ladder, with sessions lasting 55-60 minutes at 1 series of 16 repetitions.
