# Supplementary material for: The Effects of a 12-Week Training Multicomponent Exercise Program on Landing Mechanics in Recreational Athletes
Source: Healthcare (Basel). 2024 Nov 21;12(23):2327. doi: 10.3390/healthcare12232327 (PMC11641551; doi:10.3390/healthcare12232327)
Supplement: Supplementary file 1 [file healthcare-12-02327-s001.zip › healthcare-3287329-supplement S2 - Tukey test.pdf]

Supplement S2. Tukey test

Prueba de Tukey		
**VARIABLES**	**Pre EG-CG**	**Post EG-CG**
**PF1 (N/Kg)**	0.057	0.686
**PF2 (N/Kg)**	0.750	0.988
**$Force_{ML+}$ (N/Kg)**	0.690	0.500
**$Force_{ML-}$ (N/Kg)**	0.450	0.728
**$Force_{AP+}$ (N/Kg)**	0.877	0.874
**$Force_{AP-}$ (N/Kg)**	0.267	0.472
**TdEst (s)**	0.332	0.015
**I_Z (N*s)**	0.286	0.002
**I_{AP} (N*s)**	0.919	0.207
**I_{ML} (N*s)**	0.209	0.274
**ACC_{ANKLE}TOTAL (g)**	0.051	0.106
**ACC_{ANKLE}ML (g)**	0.053	0.155
**ACC_{ANKLE}AP (g)**	0.067	0.188
**ACC_{ANKLE}Z (g)**	0.091	0.335
**ACC_{KNEE}ML (g)**	0.051	0.868
**ACC_{KNEE}AP (g)**	0.055	0.307
**ACC_{KNEE}Z (g)**	0.087	0.628
